# Excitation dynamics in Photosystem I trapped in TiO_2_ mesopores

**DOI:** 10.1007/s11120-020-00730-1

**Published:** 2020-02-29

**Authors:** S. Szewczyk, R. Białek, W. Giera, G. Burdziński, R. van Grondelle, K. Gibasiewicz

**Affiliations:** 1 Faculty of Physics, Adam Mickiewicz University in Poznań, ul. Uniwersytetu Poznańskiego 2, 61-614 Poznan, Poland; 2 Department of Physics and Astronomy, Vrije Universiteit, De Boelelaan 1081, 1081 HV Amsterdam, The Netherlands

**Keywords:** Photosystem I, Time-resolved fluorescence, Transient absorption, Target analysis, Excitation dynamics, Primary charge separation, *Synechocystis*

## Abstract

Excitation decay in closed Photosystem I (PSI) isolated from cyanobacterium *Synechocystis* sp. PCC 6803 and dissolved in a buffer solution occurs predominantly with a ~ 24-ps lifetime, as measured both by time-resolved fluorescence and transient absorption. The same PSI particles deposited in mesoporous matrix made of TiO_2_ nanoparticles exhibit significantly accelerated excitation decay dominated by a ~ 6-ps component. Target analysis indicates that this acceleration is caused by ~ 50% increase of the rate constant of bulk Chls excitation quenching. As an effect of this increase, as much as ~ 70% of bulk Chls excitation is quenched before the establishment of equilibrium with the red Chls. Accelerated quenching may be caused by increased excitation trapping by the reaction center and/or quenching properties of the TiO_2_ surface directly interacting with PSI Chls. Also properties of the PSI red Chls are affected by the deposition in the TiO_2_ matrix: they become deeper traps due to an increase of their number and their oscillator strength is significantly reduced. These effects should be taken into account when constructing solar cells’ photoelectrodes composed of PSI and artificial matrices.

**Electronic supplementary material:**

The online version of this article (10.1007/s11120-020-00730-1) contains supplementary material, which is available to authorized users.

## Introduction

The essential features of any light-to-electrical current converter are efficient charge separation (creation of an electron–hole pair) and a series of fast secondary electron transfer events following the act of photon absorption. The secondary electron transfer increases the distance between the initial electron and hole and prevents from a charge recombination and an energy dissipation.

Photosystem I (PSI) is a photosynthetic pigment-protein complex in cyanobacteria, algae, and plants which fulfills these requirements. It converts photons to photoelectrons with exceptionally high quantum yield exceeding 99%. Therefore it is often tested in different biohybrid systems including solar cells (Ciesielski et al. 2008, 2010a, b; Mukherjee and Khomami 2011; Stieger et al. 2016a, b; Gunther et al. 2013; Feifel et al. 2015; Mershin et al. 2012; Gordiichuk et al. 2014; Shah et al. 2015; Yu et al. 2015; Gizzie et al. 2015; Ocakoglu et al. 2014) with the intention of invention of particularly effective device. Unfortunately, so far the efficiency of such biohybrid devices is limited. Relatively high efficiencies of some PSI-containing photoelectrodes (of the order of 0.1–0.5%) (Mershin et al. 2012; Shah et al. 2015; Yu et al. 2015) were not proven to be based on natively operating proteins.

**Fig. 1:**
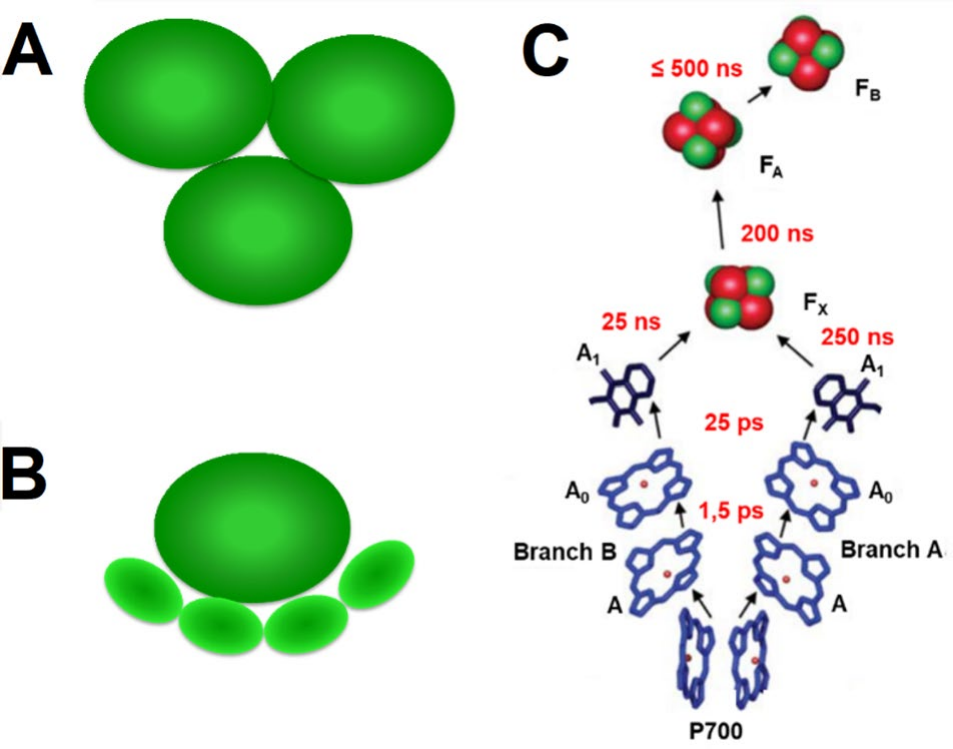
Schematic presentation of trimeric PSI structure from cyanobacteria (**a**), monomeric PSI-LHCI structure from plants (**b**), and arrangement of electron transfer cofactors in PSI core with forward electron transfer lifetimes shown in red (**c**)

PSI is a multi-subunit supercomplex composed of either three cores forming trimers (in the case of cyanobacteria) or a single core equipped with additional light-harvesting complexes (LHCI; in the case of some algae and plants) which increase the light absorption capacity of the core (Fig. 1). The structure of the PSI core from all these organisms is very similar to each other (Antoshvili et al. 2018; Fromme and Grotjohan 2008). It contains 96 chlorophyll (Chl) *a* molecules engaged in light absorption (Jordan et al. 2001). Six of these Chls together with two phylloquinones and three iron–sulfur clusters form a central part of the core called reaction center (RC) and are engaged in the charge separation and electron transfer (Fig. 1c). The remaining 90 Chls are called antenna Chls. Native operation of PSI has been determined over the last decades (Brettel 1997; Brettel and Leibl 2001; Nelson and Yocum 2006; Jensen et al. 2007; Holzwarth et al. 2006). It starts with a light absorption by the Chls followed by a fast excitation quenching caused by the primary charge separation between the RC molecules: primary donor (a chlorophyll *a* dimer labeled P700) and primary electron acceptor (either of two Chls labeled A_0_) with the transient involvement of two accessory Chls (A) (Holzwarth et al. 2006; Giera et al. 2010). These three pairs of Chls together with two phylloquinones (A_1_) are arranged in two quasi-symmetrical branches, both active in the electron transfer (Joliot and Joliot 1999; Guergova-Kuras et al. 2001; Li et al. 2006; Fairclough et al. 2003; Santabarbara et al. 2005, 2006; Ramesh et al. 2004, 2007; Giera et al. 2009). The two branches meet at the F_x_ iron–sulfur cluster, which receives an electron from A_1_ and transfers it further to two terminal electron acceptors iron–sulfur clusters, F_A_ and F_B_. The forward electron transfer events span the lifetimes from ~ 1 ps (primary charge separation or formation of the state P700^+^A_0_^−^) to ~ 500 ns (formation of the state P700^+^F_B_^−^) (Brettel and Leibl 2001) and are much faster than dissipative charge recombination reactions ranging from ~ 10 ns to ~ 100 ms, for the respective charge recombination reactions (Vassiliev et al. 1997; Kurashov et al. 2018) (~ 10 ns for P700^+^A_0_^−^ → P700A_0_, and ~ 100 ms for P700^+^F_B_^−^ → P700F_B_).

The primary charge separation is preceded by an excitation energy transfer from the antenna Chls to the RC that occurs with apparent/effective lifetime of 20–50 ps in PSI cores from various cyanobacteria (Gobets et al. 2001, 2003) and ~ 20–30 ps in PSI cores from green algae (Gibasiewicz et al. 2001; Giera et al. 2014), and is usually longer in LHCI-containing PSI preparations from algae and plants (Giera et al. 2014; Melkozernov et al. 2004; Le Quiniou et al. 2015; Wientjes et al. 2011; Slavov et al. 2008; Abram et al. 2019) due to a relatively slow energy transfer from LHCI to PSI core. The fast, 20–50 ps, excitation quenching in the PSI core is contributed by an excitation energy transfer from the core antenna system to the RC and the primary charge separation. The primary charge separation is probably a reversible reaction and occurs many times with regeneration of the excited state in the antenna system, before the excitation is definitely quenched by formation of secondary charge separated states (Giera et al. 2010; Holzwarth et al. 2005). The primary charge separation may occur even when P700 is permanently oxidized to P700^+^ (so called closed state of PSI) and the proposed primary charge separated state is A^+^A_0_^−^, which may recombine both to the excited (Chl*) and to the ground (AA_0_) state (Giera et al. 2010).

A specific feature of the PSI preparations, both cores and peripheral LHCI, is the presence of a few particularly long-wavelength Chls (called red Chls) with absorption and emission bands shifted towards the red and peaking at wavelengths longer than 700 nm (Gobets et al. 2001, 2003; Karapetyan et al. 2006; Morosinotto et al. 2003). These species originate from strong interactions between two or more Chls (Morosinotto et al. 2003; van Amerongen et al. 2000) and were hypothesized to be formed in order to (1) increase a spectral range of PSI absorption (Trissl 1993) or (2) protect PSI against an excess of light (Jensen et al. 2007; Elli et al. 2006; Carbonera et al. 2005). Their presence manifests itself in slowing down the overall excitation decay in PSI preparations (Gobets et al. 2001).

An important aspect of the application of PSI in semi-artificial devices including solar cells is the question to what extent the native mode of operation of the pigment-protein complex is preserved after its isolation from the photosynthetic membrane and its incorporation into the artificial environment. It has been demonstrated that electrodeposition of PSI cores isolated from the cyanobacterium *Synechocystis* on a flat surface of FTO glass (the glass slide covered with a thin transparent conducting layer of fluorine-doped tin oxide) resulted in a significant acceleration of the excitation decay (Szewczyk et al. 2017, 2018) and this acceleration was attributed to the dense packing of detergent-free proteins on the substrate. In the present paper we analyze how the excitation decay in PSI complexes is affected by their deposition in TiO_2_ mesopores followed by evaporation of the buffer containing detergent.

## Materials and methods

### Isolation of PSI particles

Monomeric and trimeric forms of PSI were isolated from the wild type *Synechocystis* sp. PCC 6803 strain according to the procedure described previously (Szewczyk et al. 2017).

### Preparation of TiO_2_ pastes

Preparation of the TiO_2_ pastes was the same as that one already described (Białek et al. 2018). For fluorescence measurements, the paste was prepared from 50-nm anatase nanoparticles (MKnano, 98% pure) with a procedure described by Woronowicz et al. (Woronowicz et al. 2012). Briefly, the paste was prepared in mortar by mixing TiO_2_ nanoparticles with double-distilled water with acetylacetone followed by slow addition of double-distilled water with detergent (Triton X-100).

**Fig. 2:**
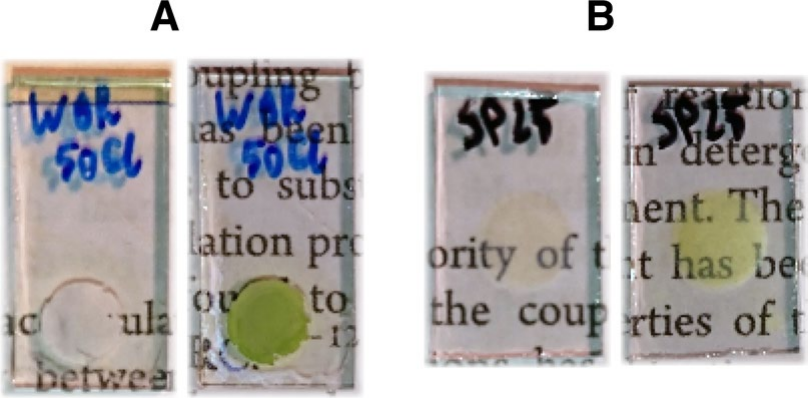
Pictures of FTO glass slides partly covered with TiO_2_ layers used for fluorescence (**a**) and absorption measurements (**b**) before and after application of the PSI suspension followed by evaporation of the solvent

TiO_2_ paste for pump-probe absorption measurements was prepared from P25 nanoparticles (PlasmaChem; 21 ± 5 nm) with a procedure described by Ito et al. (Ito et al. 2007) (with the exception that a three roller mill was not used). Briefly, the nanoparticles were mixed with water, acetic acid, ethanol, terpineol, and ethyl cellulose by subsequent treatments with a mortar, magnetic stirrer and ultrasonic horn (Sonics Vibra-Cell VCX130). An excess ethanol was evaporated using a rotary evaporator. This procedure was chosen for absorption measurements due to lower light scattering of the resultant layers compared to those obtained using the procedure by Woronowicz et al. (Fig. 2).

### Assembly of the PSI–TiO_2_ slides

Glass slides covered with FTO (Sigma-Aldrich, TEC 15) were washed in an ultrasonic bath (CT-Brand CT-432H1) sequentially in water with dish soap, double-distilled water, and ethanol for 10 min each. For fluorescence experiments, TiO_2_ paste was then deposited on the cleaned FTO glass using a doctor-blading technique (for paste formulation see above) using Scotch 3 M Magic Tape as a mask and to define layer thickness. For the pump-probe measurements, the TiO_2_ paste was deposited on the slide using a screen-printing technique with DN-HMO2 screen printer (Dyenamo) with a polyester screen of mesh count 25 (Sefar). This method gives a thinner (thus less scattering) layer than doctor-blading. This was followed by sintering in a Nabertherm 5/11 – P330 oven that was warmed up to 570 °C by 25 min and held at that temperature for the next 30 min. The active area of the TiO_2_ film was ~ 0.25 cm^2^ both for the fluorescence and absorption measurements (Fig. 2).

Purified PSI particles suspended in a buffer A containing 20 mM Bis–Tris (pH 7.0), 5 mM MgCl_2_, 5 mM CaCl_2_, 10 mM NaCl, 0.03% β-DM v/v were diluted in the same buffer to OD_680 nm, 1 cm_ = 12. Next, 4 µl of such a suspension was placed on the TiO_2_ surface and stored at 4 °C for ~ 12 h to evaporate the solvent. The PSI–TiO_2_ substrates were gently rinsed by distilled water before time-resolved experiments to wash out loosely attached proteins.

### Procedure of deposition of PS I on FTO glass

The procedure of deposition of PS I on the FTO glass was described in detail before (Szewczyk et al. 2017, 2018). Shortly, a drop of PSI solution with a reduced amount of salts and detergent was placed between two FTO electrodes, separated by a ~ 2 mm spacer. Next, the voltage of 2.5 V was applied for 5 min between the electrodes. After that, the sample was left for 12 h at 4 ℃ in order to evaporate the solvent.

### Time-resolved measurements

The time-resolved fluorescence spectroscopic experiments were carried out with a streak camera setup (Laser Center, Vrije Universiteit, Amsterdam), which was described before (Szewczyk et al. 2017). The 800-nm, 100-fs laser pulses were generated in the Ti:Sapphire laser (Coherent Vitesse) and amplified by the regenerative amplifier (Coherent RegA 9000). Their second harmonic (400 nm) obtained in the optical parametric amplifier (Coherent OPA 9400) was used for the sample excitation in all experiments. Conditions of the experiments (the energy of the pulse < 1 nJ, the repetition rate of the laser pulses—125 kHz, the spot size of the laser beam on the sample— ~ 150 µm) were chosen to exclude the singlet–singlet annihilation effects. Fluorescence signal was collected by the streak camera (Hamamatsu C5680), in three different time domains: ~ 140 ps, ~ 400 ps and ~ 1400 ps. The time resolution (temporal width of the scattered laser pulses) in those modes was ~ 3.5, ~ 6 and ~ 15 ps, respectively.

The transient absorption measurements were performed using the Helios transient absorption spectrometer (Ultrafast Systems) described earlier (Szewczyk et al. 2018). The excitation beam was generated by the Ti:Sapphire oscillator (Mai-Tai, Spectra Physics) followed by the regenerative amplifier (Spitfire Ace, Spectra Physics). The amplifier output (800 nm, 100-fs pulses) was split to generate two beams: (1) pump (405 nm) in the optical parametric amplifier (Topas Prime), and (2) probe—white-light continuum in 440–780 nm range using a sapphire crystal. The used setup enables measurements in a 2.9-ns time window with a ~ 200-fs time resolution. The energy of a single pump pulse was kept at about 15 nJ with a spot size of approximately 300 µm. Such excitation conditions minimalized singlet–singlet annihilation effects.

The PSI samples (both in solution and immobilized on TiO_2_) in both types of measurements were continuously moved to minimize effects related to an excessive irradiation. In control time-resolved fluorescence experiments (PSI in solution) the PSI suspension was placed in the rotating cuvette (~ 10 cm diameter, 2-mm thickness of the solution layer) and its concentration was set to OD_680 nm,1 cm_ = 1. For the rest of the experiments, the 2D mechanical motion controller (Newport) was used in order to move the samples in horizontal and vertical dimension. As a result, the laser beams scanned the sample in a Lissajous-like pattern. For the time-resolved absorption measurements of PSI in solution, samples were diluted in the buffer A to OD_680 nm,1 cm_ = 2 and placed in a 2 mm thick quartz cuvette.

The measurements of PSI in solution and PSI in TiO_2_ were performed without addition of any redox mediator, in order to hold similar redox conditions. Under these conditions, the PSI complexes remained in the closed state (P700 permanently oxidized to P700^+^) as evidenced by the lack of long lived absorption changes at 700 nm characteristic of formation of P700^+^ (see below).

Global and target analyses of the collected data were performed using Glotaran software (Snellenburg et al. 2012).

## Results and discussion

**Fig. 3:**
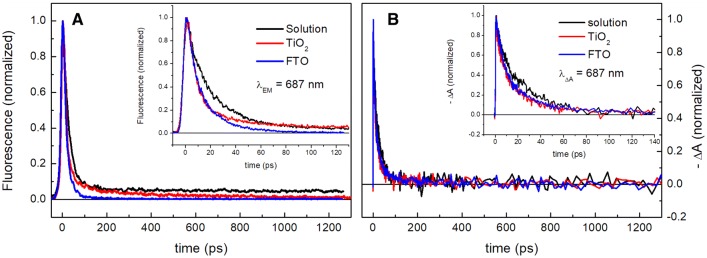
Comparison of fluorescence (**a**) and transient absorption (**b**) kinetics of PSI trimers from *Synechocystis* dissolved in a buffer solution, deposited on FTO glass, or trapped in TiO_2_ mesopores. Excitation was set at 400 nm. Note that the negative ΔA signal in panel b was inverted in order to facilitate comparison with fluorescence data presented in panel a

In Fig. 3, kinetics of fluorescence (panel a) and transient absorption (panel b) at 687 nm from three different systems containing closed PSI are compared: PSI trimers dissolved in a detergent-containing buffer, detergent-free PSI trimers electrodeposited on FTO, and PSI trimers deposited in mesoporous layer of TiO_2_. Both techniques clearly demonstrate that the excitation decay in PSI trimers trapped in TiO_2_ mesopores is significantly accelerated on a 100-ps time scale compared to the PSI trimers dissolved in a buffer. On the other hand, the effect of the accelerated decay in TiO_2_ on this time scale is similar to that one observed on PSI deposited on a flat FTO-covered glass [compare also (Szewczyk et al. 2017, 2018)]. The acceleration effect is larger in fluorescence than in transient absorption because the excitation decay is followed by a transient formation of the non-emitting short-lived charge-separated state (A^+^A_0_^−^) which slows down the decay of the photobleaching (panel b) but not the decay of emission (panel a).

The fluorescence decay in the PSI trimers in a solution contains a small long-lived component, not decaying to zero on the presented ~ 1.3-ns time scale (Fig. 3a). Such a component with a ~ 5-ns lifetime (see below) is commonly observed in virtually all PSI preparations and assigned to PSI Chls energetically uncoupled from the RC (Giera et al. 2010, 2014; Gobets et al. 2001, 2003; Gibasiewicz et al. 2001; Le Quiniou et al. 2015; Slavov et al. 2008; Holzwarth et al. 2005; Szewczyk et al. 2017, 2018, 2020; Savikhin et al. 1999). Interestingly, this phase is missing in the case of PSI-FTO system [compare (Szewczyk et al. 2017)], and is replaced by an intermediate phase slowly decaying (~ 0.5 ns, see below) for the PSI–TiO_2_ sample. The non-decaying and slowly decaying phases seen in fluorescence of PSI solution and PSI–TiO_2_ samples (Fig. 3a) are not clearly visible in the absorption kinetics (Fig. 3b) due to a poorer signal to noise ratio. However, these phases are well resolved in the global and target analysis of the absorption data (see below).

**Fig. 4:**
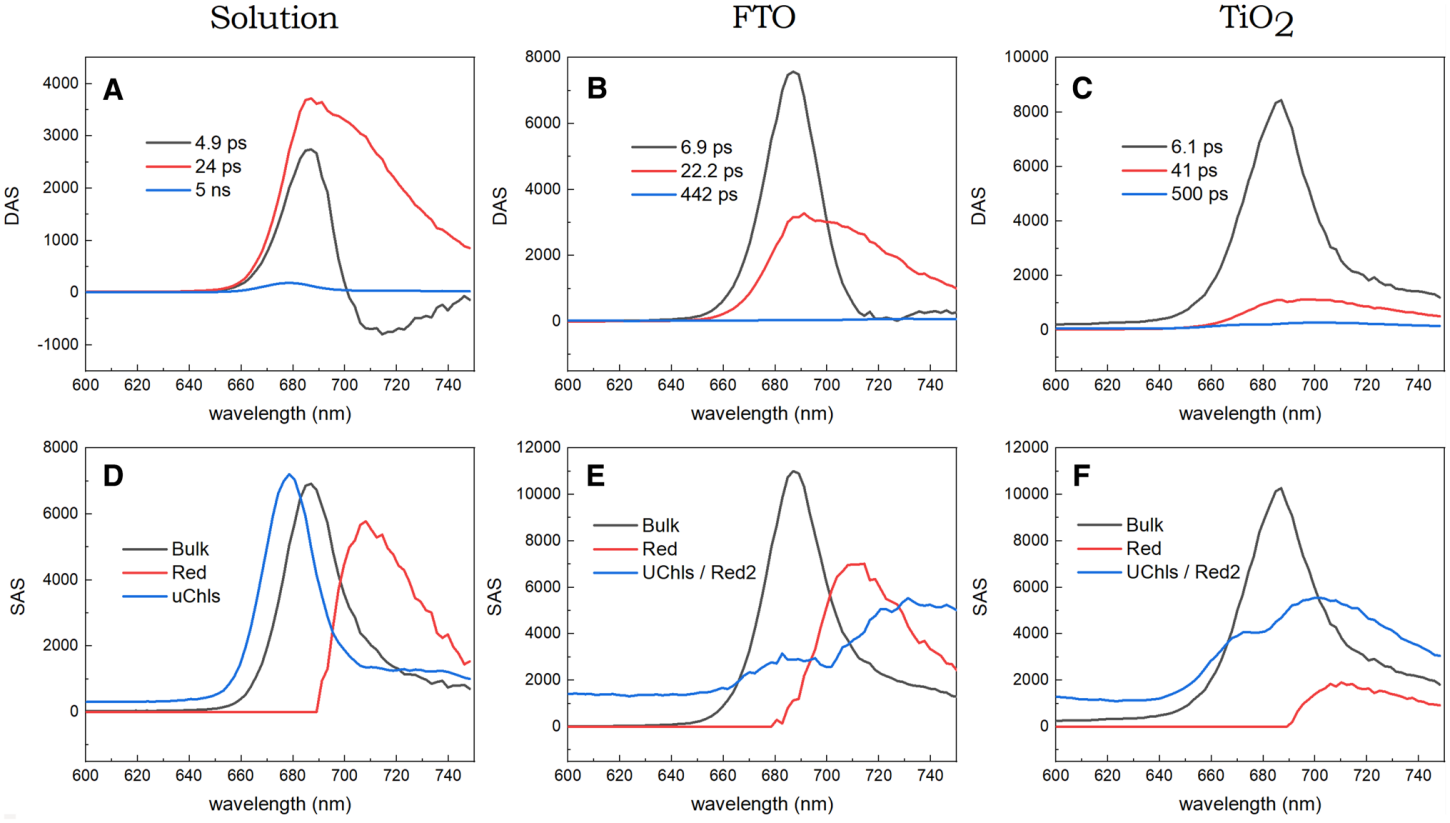
Comparison of global (DAS; **a**–**c**) and target (SAS; **d**–**f**) analysis results obtained for time-resolved fluorescence data of PSI trimers from *Synechocystis* dissolved in a buffer solution (**a**, **d**), deposited on FTO [**b**, **e**, adapted from ref. (Szewczyk et al. 2017)], or trapped in TiO_2_ mesopores (**c**, **f**). Excitation was set at 400 nm

Figure 4 presents results of global (DAS) and target (SAS) analysis of the time-resolved fluorescence measurements of closed PSI in the solution, deposited on FTO (adapted from ref. Szewczyk et al. 2017), and in the TiO_2_ mesopores. In all the cases, the global analysis yielded two sub-100-ps components of similar spectral positions, shapes, and lifetimes (4.9 ps and 24 ps in solution, 6.9 ps and 22 ps on FTO, 6.1 ps and 41 ps in TiO_2_) but of very much different relative amplitudes (panels a–c). In the solution, the 4.8-ps component clearly describes the energy equilibration process between bulk Chls emitting at ~ 687 nm and red shifted Chls emitting at ~ 715 nm. The larger amplitude of the positive part of this DAS than its negative part reveals a minor contribution of the overall excitation decay occurring on the same time scale. On the other hand, the 24-ps DAS peaking at ~ 687 nm is of a higher amplitude, is much broader and asymmetric with a bump above 700 nm on a long-wavelength slope of the band, and is positive in the whole spectral range. Therefore, this DAS is assigned to the decay of the excitation equilibrated over the bulk and long-wavelength Chls. In contrast to the PSI in solution, the short, 6.1-ps DAS strongly dominates in the overall decay of excitation in the PSI–TiO_2_ system and no negative amplitudes attributable to excitation equilibration are seen. The 41-ps DAS is broadened towards the red similarly as the 24-ps DAS for PSI in solution but is of small amplitude. Apparently, most of the excitation decays from the bulk Chls non-equilibrated with the red-shifted Chls, and only a minority of excitation is equilibrated between the bulk and red Chls and decays with 41-ps lifetime. The relative amplitude of the 22-ps DAS in the PSI-FTO system is intermediate between the corresponding DAS-es for PSI in solution and in TiO_2_. In addition to the sub-100-ps DAS, a 5-ns DAS (peaking at ~ 679 nm) as well as a 0.5-ns DAS (peaking at ~ 670 and at ~ 700 nm) of very small amplitudes were resolved in PSI solution and PSI–TiO_2_ systems, respectively, and are discussed below.

**Fig. 5:**
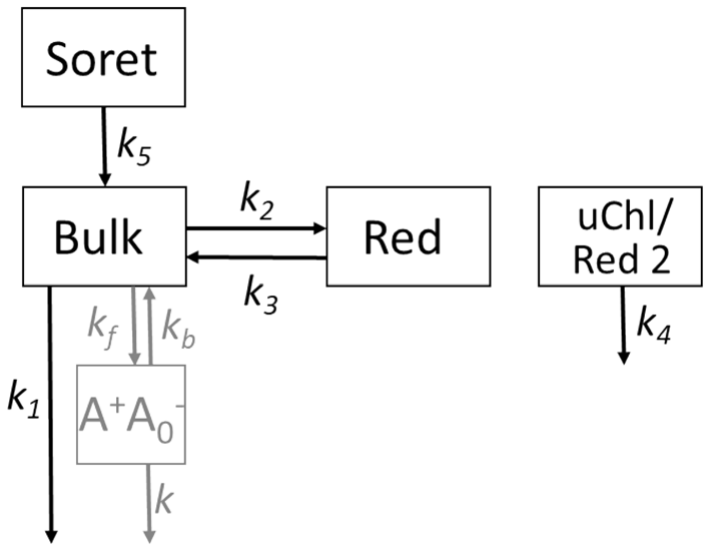
In black: compartmental model used in target analysis of results of time-resolved fluorescence and time-resolved absorption shown in Figs. 4 and 6 (SAS). In gray: rate constants (*k*_*f*_, *k*_*b*_, and *k*) affecting the value of *k*_1_; in the closed RCs, *k* depicts charge recombination reaction (A^+^A_0_^−^ → AA_0_)

The features seen in the DAS are reflected in the results of target analysis (TA). The compartmental model used in TA is shown in Fig. 5 and the resultant rate constants are collected in Table 1. Three compartments were taken into account to model the fluorescence data of PSI in solution, PSI on FTO, and PSI in TiO_2_: bulk Chls, red Chls being in equilibrium with bulk Chls, and uncoupled Chls (uChls) (mixed in one compartment with a second pool of red Chls in the case of PSI–TiO_2_ system—see below). In the case of the transient absorption data, characterized by about ten times better temporal resolution than the fluorescence data, an additional compartment, “Soret”, was introduced in order to model sub-picosecond processes of (1) the relaxation from the initially excited Soret state to the Q_y_ state and (2) the excitation relaxation between relatively high-energy (blue shifted) Chls and bulk Chls.

**Table 1:** Parameters obtained from global and target analysis of time-resolved fluorescence and transient absorption data obtained for the closed PSI complexes from *Synechocystis* in solution, deposited on FTO, and incorporated into the TiO_2_ mesopores

		Trimers						Monomers					
		Fluorescence			Absorption			Fluorescence			Absorption		
		Solution	FTO^a^	TiO_2_	Solution	FTO^b^	TiO_2_	Solution^a^	FTO^a^	TiO_2_	Solution^b^	FTO^b^	TiO_2_
Global analysis	*τ*_1_ [ps]	–	–	–	0.4	0.7	0.4	–	–	–	0.6	0.6	0.3
	*τ*_2_ [ps]	4.9	6.9	6.1	3.2	5.1	4.4	5.3	6.5	5.4	4.2	4.2	4.3
	*τ*_3_ [ps]	24	22	41	24	24	25	24.5	24.5	29	25	22	30
	*τ*_4_ [ps]	5 000	442	500	5 000	454	900	5 000	227	500	5 000	625	1 600
Target analysis	1/*k*_1_ [ps]	15.5	10.5	10.1	17.5	14.3	14.8	16.1	10.3	9.6	20.0	13.1	16.9
	1/*k*_2_ [ps]	19.6	36.7	21.1	14.4	22.0	15.6	23.3	30.0	18.5	22.3	15.4	13.6
	1/*k*_3_ [ps]	7.8	14.1	25.4	4.4	7.9	7.4	8.1	15.4	16.3	5.3	7.2	7.6
	1/*k*_4_ [ps]	5 000	442	500	5 000	454	900	5 000	228	500	5 000	626	1 600
	1/*k*_5_ [ps]	–	–	–	0.4	0.7	0.4	–	–	–	0.3	0.6	0.3
	$\Delta {G}_{b-r}^{0}$ [meV]	− 23	− 25	+ 5	− 30	− 26	− 19	− 27	− 17	− 3	− 37	− 20	− 15
	$\Delta {H}_{b-r}^{0}$ [meV]	+ 61	+ 59	+ 61	–	–	–	+ 62	+ 52	+ 63	–	–	–
	*N*_r_	4	4	10	–	–	–	3	6	7	–	–	–

*τ*_*i*_—lifetimes obtained from global analysis; *k*_*i*_—rate constants obtained from the target analysis; $\Delta {G}_{b-r}^{0}={G}_{b}^{0}-{G}_{r}^{0}=-{k}_{B}Tln\frac{{k}_{3}}{{k}_{2}}$ is the standard (Gibbs) free energy difference between bulk (${G}_{b}^{0}$) and red (${G}_{r}^{0}$) compartments in Fig. 5 (${k}_{B}$—Boltzmann constant, T—absolute temperature); $\Delta {H}_{b-r}^{0}=hc(\frac{1}{{\lambda }_{b}}-\frac{1}{{\lambda }_{r}})$ is the standard enthalpy difference between bulk and red Chls states (*h*—Planck constant, *c*—speed of light in vacuum, *λ*_*b*_ and *λ*_*r*_—respective wavelengths of the bulk and red Chls SAS at their maxima—see Fig. 4; see (Szewczyk et al. 2017, 2018) for further details). *N*_*r*_—effective number of red Chls estimated on the basis of $\Delta {G}_{b-r}^{0}$ and $\Delta {H}_{b-r}^{0}$

^a^Values taken from(Szewczyk et al. 2017)

^b^Values taken from (Szewczyk et al. 2018). In the references (Szewczyk et al. 2017, 2018), 3 mM potassium ferricyanide was added to the solution of PSI in order to keep P700 oxidized

The spectra of the three emitting compartments resulting from TA for the PSI in solution are shown in Fig. 4d and are very similar to those published before (Szewczyk et al. 2017). A maximum of bulk Chls emission is at ~ 687 nm, a maximum of red Chls emission—at ~ 710 nm, and a maximum of the uChls—at ~ 679 nm. After introducing PSI into the TiO_2_ mesopores (Fig. 4f), the bulk Chls compartment remains almost unaffected (except for a modest broadening), the red Chls compartment preserves its shape but its amplitude is reduced by a factor of ~ 3, and the uChls compartment is characterized by two bands—one typical for uChls (peaking at ~ 670 nm) and another one, red-shifted to ~ 700 nm. The reduced amplitude of the red Chls SAS in the TiO_2_ mesopores may be explained by a reduced oscillator strength caused by the interaction of PSI complexes with the TiO_2_ substrate. A similar, although significantly less pronounced effect was observed before for PSI tightly deposited on a flat surface of FTO glass (Fig. 4e). Also the double-peak structure of the similar 0.5-ns SAS was observed before for PSI on FTO, albeit with maxima at longer wavelengths of ~ 680 and ~ 730 nm (Szewczyk et al. 2017) (Fig. 4e).

**Fig. 6:**
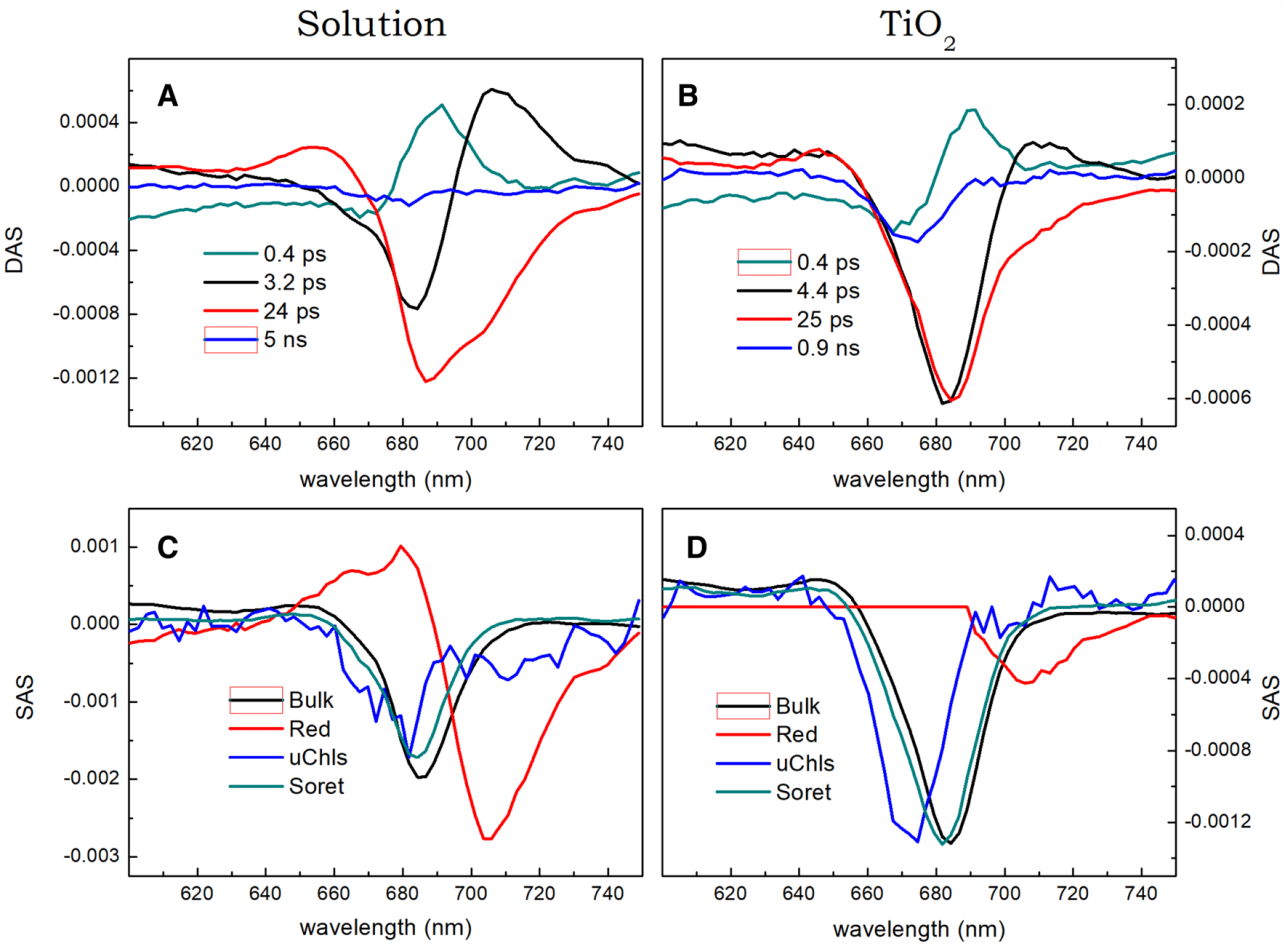
Comparison of global (DAS; **a**, **b**) and target (SAS; **c**, **d**) analysis results obtained for transient absorption data of PSI trimers from *Synechocystis* either dissolved in a buffer solution (**a**, **c**) or trapped in the TiO_2_ mesopores (**b**, **d**). Excitation was set at 400 nm

In Fig. 6, DAS and SAS obtained from an analysis of time-resolved absorption measurements are compared. For both PSI samples, the sub-picosecond DAS (Fig. 6a, b) describes a mixture of Soret to Q_y_ internal conversion and the blue to bulk Chls energy transfer—mixing of both processes is reflected by the non-conservative shape of the respective DAS with smaller negative part (at ~ 670 nm) and larger positive part (at ~ 690 nm). The second DAS of 3.2–4.4 ps depicts, similarly to the respective fluorescence DAS (Fig. 4a, c), mostly excitation energy transfer from bulk to red Chls in the case of PSI in solution, and mostly excitation decay in the case of PSI–TiO_2_ sample. The difference between these two DAS reflects faster decay of photobleaching/stimulated emission signal in PSI–TiO_2_ than in PSI in solution seen in Fig. 3b. The third DAS, of 24–25 ps lifetime, corresponds to the 24–41-ps DAS from Fig. 4a, c. The effect of introducing PSI into the TiO_2_ mesopores on this component is similar to that observed in fluorescence (Fig. 4a, c) albeit less dramatic—the relative amplitude of this phase decreases. The smaller effect seen in absorption may be explained by contribution from the photobleaching of the charge separated dark state A^+^A_0_^−^ which is absent in the fluorescence data. The slowest DAS decays within 5 ns for PSI in solution and in 0.9 ns in PSI–TiO_2_ and similarly as in fluorescence data is assigned to, respectively, uncoupled or weakly coupled Chls as suggested by long lifetimes and blue-shifted position of the peaks (to ~ 674–680 nm).

TA of the absorption data (Fig. 6c, d) reveals mostly the same features as in the case of the fluorescence data. The signal from the bulk Chls peaks in both samples at ~ 685 nm (slightly blue shifted, due to Stokes shift, relative to the bulk Chls emission peaking at ~ 687 nm). Maximum of the red Chls pool is at ~ 705 nm in both samples (again ~ 5-nm blue shifted relative to the fluorescence SAS in Fig. 4d, f). Uncoupled Chls bands peak at ~ 675–680 nm, towards the blue relative to the bulk Chls, as expected. In contrast to the fluorescence data (Fig. 4f) the uChls compartment in absorption (Fig. 6d) does not possess any red Chls contribution. The reason for this minor difference remains unclear.

TA of the absorption data yields also a spectrum of the “Soret” compartment. It should be interpreted as a mixture of two very similar spectra. One is a pure Q_y_ photobleaching spectrum of bulk Chls without significant contribution of stimulated emission (spectrum of the bulk Chls before the internal conversion from the Soret to Q_y_ band took place). Another one is a Q_y_ photobleaching spectrum of relatively blue-shifted bulk Chls with a contribution of stimulated emission (after internal conversion). In the solution (Fig. 6c), both spectra are represented and therefore the amplitude of the “Soret” spectrum is smaller than the amplitude of the “Bulk” spectrum. In TiO_2_ (Fig. 6d), the “Soret” and “Bulk” spectra are of similar amplitudes and the 0.4-ps DAS (Fig. 6b) is more conservative than in solution (Fig. 6a). These features indicate that the “Soret” spectrum in Fig. 6d is dominated by the latter component—blue Chls photobleaching plus stimulated emission. In both cases (Fig. 6c, d), a similar shape of the “Soret” and “Bulk” spectra together with a slight red-shift of the latter one are understandable and expected.

The values of the reciprocals of the rate constants shown in Fig. 5, obtained from the TA are collected in Table 1 both for the trimeric and monomeric PSI complexes in the solution, on FTO, and in the TiO_2_ mesopores. The values obtained for the monomeric PSI are very much consistent with those for the trimeric PSI and therefore, only the latter ones will be discussed in detail. The major observation explaining the acceleration of the fluorescence and absorption signal decay (Fig. 3) following deposition of PSI in the TiO_2_ mesopores is shortening of 1/*k*_1_ from 15.5 to 10.1 ps in fluorescence (corresponding to ~ 50% increase of *k*_1_) and from 17.5 to 14.8 ps in the absorption experiment. Longer lifetimes in the case of the absorption are consistent with the involvement of the state A^+^A_0_^−^ (detected in the absorption but not in the fluorescence) which appears in the closed RCs and decays slightly slower than the excited state (Giera et al. 2010). Expected involvement of the dark state A^+^A_0_^−^ in excitation decay and its influence on the rate *k*_1_ is presented in Fig. 5 by the gray part of the scheme.

Acceleration of the process depicted by the rate *k*_1_ in response to incorporation of the PSI complexes into TiO_2_ is responsible for quenching of the large majority (~ 70% = $\frac{{k}_{1}}{{k}_{1}+{k}_{2}}100\%$) of bulk Chls excitation before equilibration between the bulk and red Chls occurs. Similar acceleration of the bulk Chls quenching in PSI was observed before for PSI trimers and monomers deposited on silan- or FTO-covered glass and it was concluded to be caused by the dense PSI packing on the flat surface (Szewczyk et al. 2017, 2018). Such a dense packing was possible due to the removal of detergent from the PSI solution by dialysis, that in turn promoted side-side interaction between the PSI proteins. However, the solution of the PSI complexes deposited on TiO_2_ contained detergent. Moreover, the mesoporous structure of TiO_2_ gives enough free space for accommodation of all applied PSI proteins. For these reasons no crowding effect in the case PSI–TiO_2_ system is expected. Therefore we propose that the reason for the accelerated quenching is adhesion of PSI complex to solid substrate inducing (1) increased excitation trapping by RC and/or (2) excitation quenching by the TiO_2_ surface directly interacting with the PSI Chls.

**Fig. 7:**
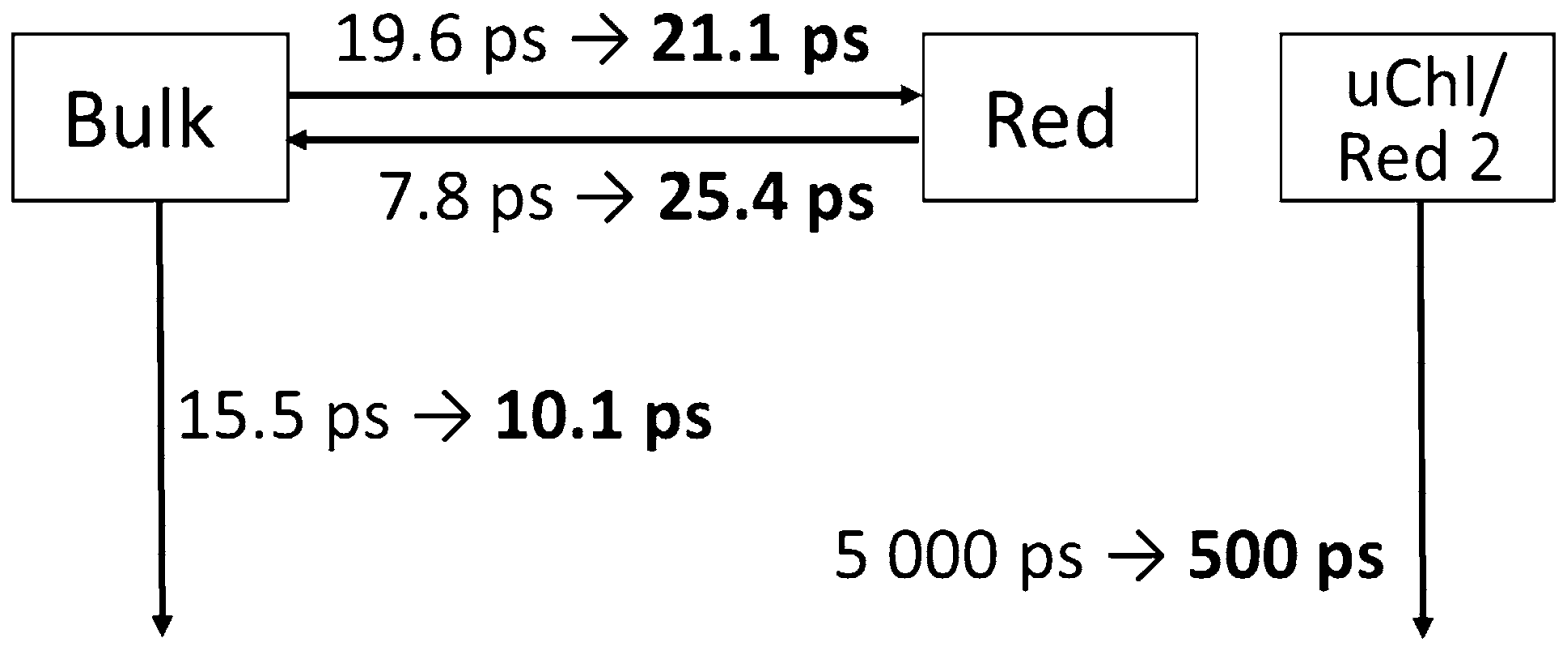
Comparison of the molecular lifetimes resulting from the target analysis of the fluorescence measurements of PSI trimers in solution (regular font) and incorporated into the TiO_2_ mesopores (in bold; compare Table 1). Distribution of the initial excitation was ~ 92%—Bulk, ~ 4%—Red, ~ 4% uChl/Red 2

Another effect of the PSI interaction with the interior of the mesopores is significant ~ 1.5–3-fold increase in the lifetime 1/*k*_3_ (Table 1; Figs. 5 and 7). This effect, together with less significant modifications of the 1/*k*_2_ values, reveals a shift in the Bulk ↔ Red equilibrium towards the red. Apparently, the free energy of red Chls shifts down relative to bulk Chls as a result of the PSI deposition in mesopores. This is reflected by different free energy gap, ${\Delta G}_{b-r}^{0}$, between bulk and red Chls in PSI in TiO_2_ than in solution (Table 1). This shift partly counteracts the acceleration of the excitation decay caused by shortening of the lifetime 1/*k*_1_.

The reason why the free energy gap, ${\Delta G}_{b-r}^{0}$, increases following the PSI deposition in TiO_2_ pores, despite almost unchanged spectral position of the bulk (~ 687 nm) and red (~ 708–710 nm) Chls (compare Fig. 4d, f), may be increased number of red Chls in the PSI–TiO_2_ system. As can be seen in Table 1, the free enthalpy difference between bulk and red Chls, ${\Delta H}_{b-r}^{0}$, calculated from the peak positions of the bulk and red Chls SAS, is identical for PSI in solution and in TiO_2_. On the basis of ${\Delta G}_{b-r}^{0}$ and ${\Delta H}_{b-r}^{0}$, it is possible to estimate the number of red Chls per PSI monomer, assuming the total number of ~ 100 Chls per PSI monomer, from the following expression derived from the equations given in references (Szewczyk et al. 2017, 2018):

$$N_{r} = \frac{100}{{1 + e^{{\frac{{\Delta H_{b - r}^{0} - \Delta G_{b - r}^{0} }}{{k_{B} T}}}} }} .$$

The resulting number of red Chls per monomeric PSI deposited in TiO_2_ (10 and 7 for PSI trimers and monomers, respectively) is ~ 2.5-fold larger than the number of red Chls in PSI in solution (Table 1). In consequence, the decreased oscillator strength of the red Chls (see above) is compensated by their increased number. This compensation could be the reason why no significant differences between the steady-state absorption spectra of PSI in solution and in TiO_2_ are observed in the long-wavelength region, > 680 nm (Fig. S1 in the Suppelmentary Information). A similar, twofold increase in the number of red Chls was reported before for PSI monomers deposited on FTO (Szewczyk et al. 2017).

A separate question is what is the nature of the additional red Chls. We hypothesize that the new red states may come from groups of the PSI (peripheral?) Chls which in the solution are bulk Chls weakly interacting with each other, but forced by the interaction with the TiO_2_ substrate, they become excitonically coupled systems.

Regarding the large decrease in the oscillator strength of the red Chls after the PS I deposition on TiO_2_ we speculate that it may be caused by redistribution of the oscillator strength from the low-energy (long wavelength, > 700 nm) to the high-energy excitonic bands (of interacting Chls forming the red states), with the latter ones spectrally overlapping with the bulk Chls. Thus, the total oscillator strength of the excitonically interacting Chls does not need to be affected by the interaction between PSI and TiO_2_. It is known that the distribution of the oscillator strength between excitonic states critically depends on the mutual orientation of the interacting molecules (van Amerongen et al. 2000). The new red states appearing as a result of the interaction between PSI and TiO_2_ may come from the excitonically interacting Chls whose mutual orientation privileges high contribution of the higher excitonic state(s) at the expense of the oscillator strength of the low energy band (> 700 nm).

Finally, one should consider the potential influence of the elevated salt concentration in the vicinity of the PS I complexes immobilized in the TiO_2_ pores after the evaporation of the solvent. First, the immobilized PSI particles are expected to be covered with a layer of detergent molecules protecting the proteins from the direct contact with the salt. Secondly, the slow evaporation should result in crystallization of the salt. Under such assumptions, no ions should strongly interact with the protein surface and no influence of the elevated salt concentration on the excitation dynamics in PS I trapped in the TiO_2_ mesopores is expected.

## Conclusions

Incorporation of the PSI cores in the TiO_2_ matrix leads to three clear effects which are summarized in Fig. 7 and affects all three compartments considered in the model: bulk, red, and uncoupled Chls. (1) Excitation of bulk Chls decay is accelerated by ~ 50%. A similar acceleration was previously observed for PSI cores deposited on the FTO glass (Szewczyk et al. 2017). (2) The red Chls become deeper traps and their oscillator strength is reduced by a factor of ~ 3. (3) The lifetime of the uncoupled Chls decreases and their spectrum becomes complex with an additional band in the red. These effects should be taken into account when constructing PSI-containing semi-artificial biohybrid materials.

## Electronic supplementary material

Below is the link to the electronic supplementary material.

Supplementary file1 (DOCX 119 kb)
