# Supplementary material for: Excitation dynamics in Photosystem I trapped in TiO2 mesopores
Source: Photosynth Res. 2020 Feb 29;144(2):235–45. doi: 10.1007/s11120-020-00730-1 (PMC7203582; doi:10.1007/s11120-020-00730-1)
Supplement: Supplementary file 1 — Supplementary file1 (DOCX 119 kb) [file 11120_2020_730_MOESM1_ESM.docx]

**Supplementary Information**

Excitation dynamics in Photosystem I trapped in TiO_2_ mesopores

S. Szewczyk^1^, R. Białek^1^, W.Giera^1^, G. Burdziński^1^, R. van Grondelle^2^, and K. Gibasiewicz^1^

^1^ Faculty of Physics, Adam Mickiewicz University in Poznań, ul. Uniwersytetu Poznańskiego 2, 61-614 Poznań, Poland

2 Department of Physics and Astronomy, Vrije Universiteit, De Boelelaan 1081,1081 HV Amsterdam, The Netherlands

# Steady-state absorption spectra

In Fig. S1, the steady-state absorption spectra of the samples used in the transient absorption (SP25+PSI) and time-resolved fluorescence (W50+PSI) measurements are shown. Due to the low absorption of the PSI complexes (particularly low in the case of the SP25+PSI samples; A(678 nm, 1 cm) ≈ 0.05) accompanied by strong scattering and UV absorption by the mesoporous TiO_2_ substrate (particularly strong in the case of the W50 substrate) the steady-state absorption spectra of the PSI-TiO_2_ systems are highly uncertain. The PSI spectra have been measured using the integration sphere. They needed a correction - subtraction of the absorption spectrum of the TiO_2_ substrate without PSI. Since the spectra of the substrates differed from sample to sample, the effect of the correction was not perfect. However, if one focuses attention on the red edge of the Q_y_ band (at > 680 nm) no strong differences in the shape of the PSI absorption can be seen when comparing the PSI complexes in the solution and those deposited onto the TiO_2_ substrate.


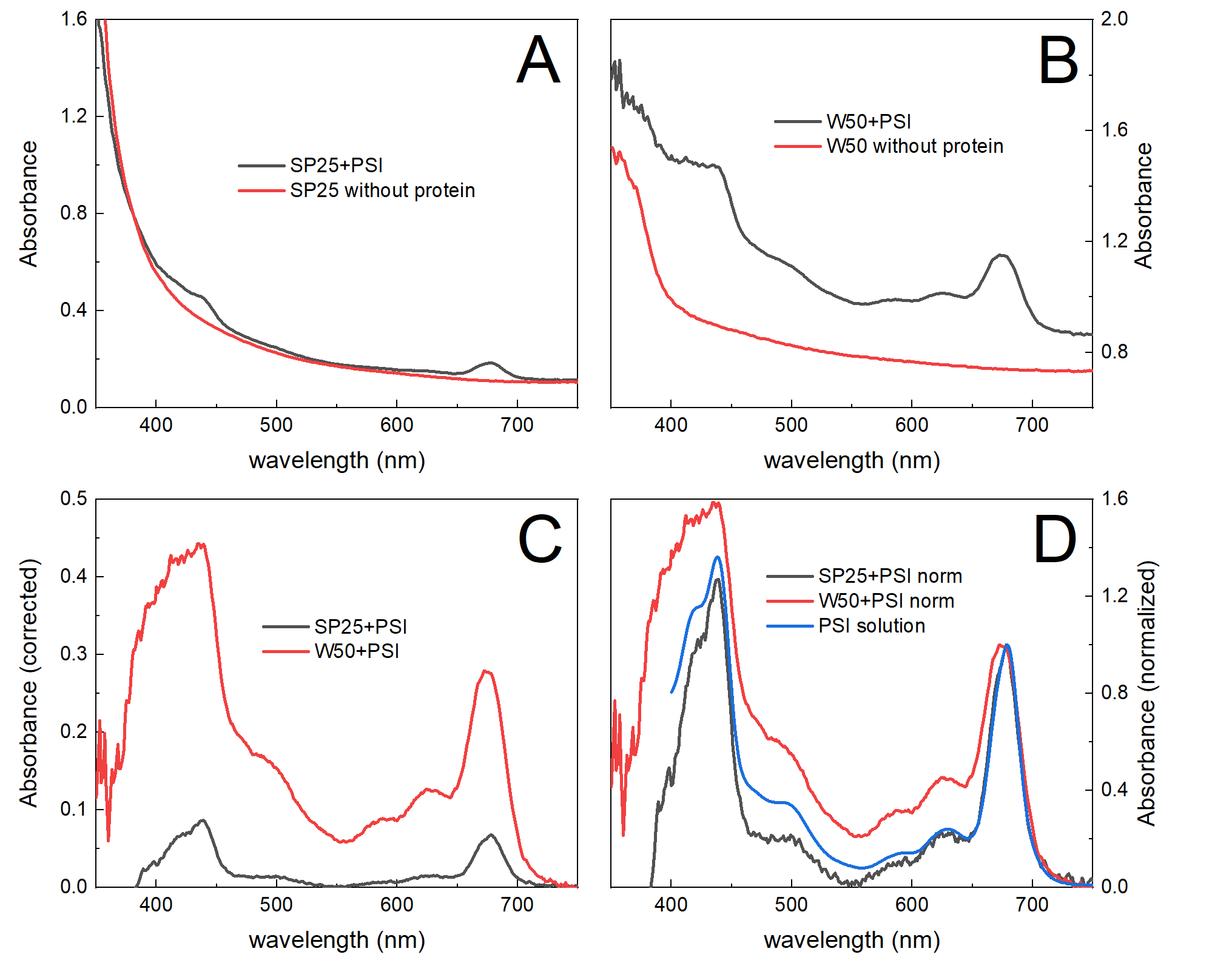


Fig. S1. Steady-state absorption spectra of the PSI complexes deposited onto the mesoporous TiO_2_ substrate. A, B – raw spectra for PSI-TiO_2_ samples (black) and TiO_2_-only reference samples (red) measured for two different TiO_2_ substrates: more transparent (SP25, panel A) and less transparent (W50, panel B); C – corrected spectra of PSI from the systems shown in panels A and B, with subtracted absorption of the TiO_2_-FTO substrate; D – the same spectra as in panel C but normalized at the maximum of the Q_y_ absorption band; additionally, a normalized absorption spectrum of the PSI complexes in solution is shown.
